# Progress and Challenges of Three-Dimensional/Two-Dimensional Bilayered Perovskite Solar Cells: A Critical Review

**DOI:** 10.3390/nano15120876

**Published:** 2025-06-06

**Authors:** Ashraful Hossain Howlader, Ashraf Uddin

**Affiliations:** School of Photovoltaic and Renewable Energy Engineering, UNSW Sydney, Sydney, NSW 2033, Australia; a.howlader@unsw.edu.au

**Keywords:** 3D/2D perovskite, aliphatic ammonium, aromatic ammonium, surface passivation, bulk passivation

## Abstract

Three-dimensional/two-dimensional bilayered perovskite solar cells have recently become popular for ensuring high efficiency and promising long-term stability. The 3D/2D bilayered perovskite thin film is mainly used in regular (n-i-p)-type perovskite solar cells. In this review, our discussion also focuses on the regular kind of perovskite solar cells. In a 3D/2D bilayered perovskite thin film, the 2D perovskite layer works as a capping layer on top of the 3D perovskite thin film. The 2D capping layer heals the surface and bulk defects of the 3D perovskite thin film. The 2D layer interfaces between the 3D perovskite and hole transport layers. The 2D layer also acts as a shield against moisture and heat. This layer also inhibits ion migration between layers (3D perovskite and back contact). This review lists and investigates different organic precursors deposited as a 2D capping layer on top of the 3D perovskite thin film to explore their impact on the solar cell’s efficiency and stability. The possible challenges and remedies in growing a 2D capping layer on top of the 3D perovskite thin film are also discussed.

## 1. Introduction

After silicon solar cells, among different next-generation solar cells, perovskite solar cells (PSCs) have the best efficiency output [1,2,3]. The latest certified record efficiency is more than 25%, making them a strong competitor in the future solar market [4]. Their low-cost and earth-abundant fabrication ingredients and low-temperature solution-processed fabrication ability make them commercially attractive for roll-to-roll production [5]. Various aspects of perovskites make them attractive for solar energy harvesting, especially their optoelectronic properties. Ambipolar charge transport [6], long charge diffusion length [7,8], shallow low defect density [9,10], and high defect tolerance [11,12,13] are a few to mention. However, we still need to solve some challenges in commercializing PSCs. One of the main challenges is the creation of vacancies and pinholes during the solution-processed fabrication. Perovskite has a low activation energy for ion migration, and the vacancies are the primary route [14,15]. Hysteresis, the inherent characteristic of perovskite, is thought to be the result of ion migration [16,17,18]. Again, phase instability, another intrinsic characteristic of perovskite, is closely related to ion migration [19,20]. Under environmental factors like light, water, and heat, the perovskite changes to an energetically favorable non-perovskite phase where the lattice structure collapses [21].

Again, mixed-halide perovskite is more popular than single-halide perovskite. The most common perovskite for PSCs is iodine (I)-based. A small amount of bromine (Br) doping enhances the stability of the perovskite [22]. A small amount of chlorine (Cl) enhances the diffusion length of the perovskite [23]. While different halogens are mixed, there is a radius mismatch between the halogens [24]. The radius mismatch creates local stress and strain in the crystal lattice, specifically in the octahedron. This encourages the deformation of the crystal lattice and frees the halogens. The free halogens diffuse through atomic vacancies and generate different, unique phenomena. For example, in the radius mismatch between Cl and I in chloride–iodide perovskite in PSC, the migrated Cl forms the SnCl_2_ layer at the interface of the perovskite/SnO_2_ electron transport layer (ETL). The SnCl_2_ layer works as a self-formed passivation layer at the buried interface [25].

From the above discussion, it is clear that vacancies in the bulk of the perovskite thin film need to be passivated to reduce ion migration in perovskite. Another fact is that halide ions are the most mobile among different ions, and the halogen concentration variation across the thin film is due to ion migration. It would be good if the perovskite’s bulk could be passivated with halide ions. Again, the surface and interfaces of the perovskite with the hole transport layer (HTL) contain many defects like grain boundaries and voids, which should also be passivated. Meanwhile, long or bulky organic salts have become popular to passivate the perovskite’s bulk and surface. These organic salts have long or bulky cations that cannot diffuse into the bulk or remain on the surface, while the loosely bonded halogen atoms easily diffuse into the bulk. In some cases, the long or bulky organic cations react with unreacted ions on the surface of the perovskite and form a 2D perovskite on the 3D perovskite. The 2D perovskite acts as a protective capping layer on the 3D perovskite thin film. This strategy has attracted attention due to superior moisture resistance and the hydrophobicity of the long or bulky spacer cations. The stability of the 2D perovskite-based PSC is better than that of the 3D perovskite-based PSC, while the efficiency of the 3D perovskite-based PSC is better than that of the 2D perovskite-based PSC. A combined 3D/2D perovskite can leverage the two parameters better. However, the 2D perovskite has a problem in insulating the photocurrent. To overcome this problem, the deposited 2D perovskite layer is made too thin. Thus, better stability can be achieved without compromising efficiency. Using this strategy, many works have been carried out with promising results. The promising results encourage us to explore 3D/2D perovskite-based solar cells with various forms of long or bulky organic cations and prepare a review paper.

Various works in the literature focus on 3D/2D perovskite-based solar cells. The 2D perovskite capping layer can be developed at the interfaces of the HTL with the 3D perovskite layer. Again, the 2D perovskite capping layer can be developed at the buried or top interfaces with the 3D perovskite layer. It is observed that the band alignment of the 2D perovskite layer with the HTL is more suitable. At the same time, identifying and characterizing the growth and formation of the 2D perovskite capping layer of the buried interface is difficult. Therefore, most of the literature focuses on the 2D perovskite capping layer between the top interface of the 3D perovskite layer and the HTL. This review focuses on 3D/2D PSCs, where the extremely thin 2D perovskite is deposited between the 3D perovskite thin film and HTL.

## 2. Organic Precursors

**Figure 1:**
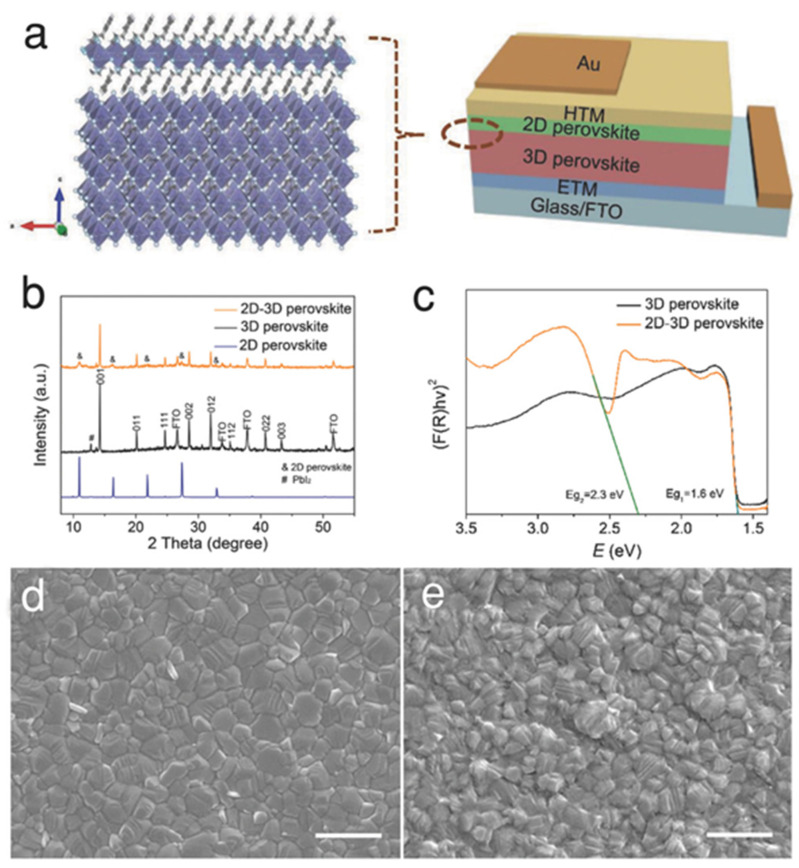
(**a**) Schematic illustrations of 3D/2D bilayered perovskite crystal structure and the device architecture. (**b**) XRD patterns of the 3D, 2D, and 3D/2D perovskite thin films. (**c**) Measured ultraviolet–visible spectra of the 3D and 3D/2D perovskite thin films. (**d**) SEM image from the surfaces of the 3D perovskite thin film (the scale bar is 1 µm). (**e**) SEM image from the 3D/2D perovskite thin film (the scale bar is 1 µm). Reprinted with permission from Ref. [26].

Recently, the growth of a 2D capping layer on a 3D perovskite thin film has become a promising method for obtaining high efficiency while maintaining stability. Figure 1a shows the crystalline structure of the 3D/2D bilayer perovskite and a PSC made of it. The bilayer perovskite comprises an extremely thin layer of 2D perovskite on a 3D perovskite thin film. The extremely thin perovskite layer behaves like a 2D perovskite capping layer, which can be identified with various characterization tools such as x-ray diffraction (XRD), ultraviolet–visible (UV-vis) spectroscopy, etc. The bilayer perovskite shows different characteristic peaks corresponding to the 2D perovskite in the XRD pattern (see Figure 1b). The bilayer perovskite shows two sharp changes in absorption, one corresponding to the 2D perovskite (see Figure 1c). From the schematic structure of the PSC (see Figure 1a), we see that the extremely thin perovskite layer also works as an interface layer between the active perovskite layer and HTL. Usually, a conductive material is preferable for this purpose. As the large organic cations are slightly non-conductive, they reduce the device current at the interface of the perovskite and HTL. Therefore, the 2D layer is thin to yield the charge tunneling effect. Although the large organic cations are considered to create a charge extraction barrier, the halide counterparts offer a promising role in the passivation of halide vacancies in the bulk of the perovskite thin film. The large organic cation also influences the stability of the PSC. In most cases, the cation of these organic perovskite salts remains on the surface of the 3D perovskite thin film and passivates the grain boundaries. The surface passivation effect can be visualized with microscopy images. The scanning electron microscopy image from the 3D perovskite surface shows clear grain boundaries, whereas the 3D/2D surface shows almost no grain boundaries (see Figure 1d,e).

**Figure 2:**
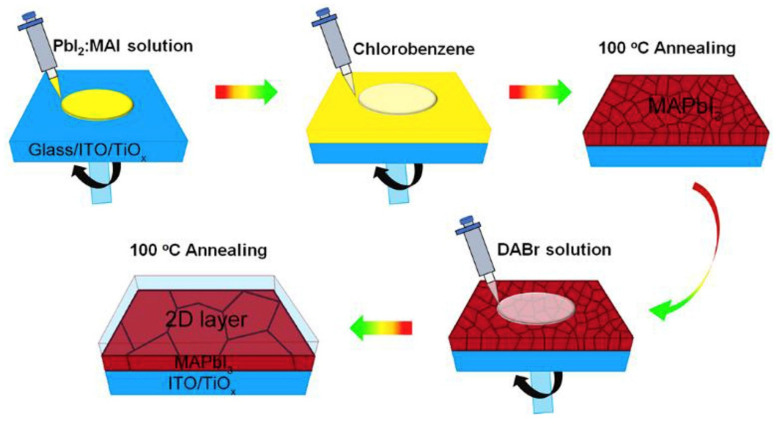
Schematic presentation of the processing steps for the growth of extremely thin 2D perovskite film on top of 3D perovskite thin film using the spin coating method. Reprinted with permission from Ref. [27].

The most popular fabrication process of 3D/2D bilayered perovskite heterostructures is the in situ growth of an extremely thin 2D perovskite layer on a 3D perovskite thin film. The organic salts are dissolved in isopropyl alcohol (IPA) solvent and deposited on the prefabricated 3D perovskite thin film via spin coating. In spin coating, as shown in Figure 2, the organic salt solution is dropped onto the prefabricated 3D perovskite thin film. The thickness of the 2D capping layer can vary with the molarity of the solution. However, the thickness of the 2D capping layer can be further changed by varying the spinning speed. The excess PbI_2_ in the 3D perovskite thin film readily reacts with the organic salts and forms the 2D perovskite capping layer on the surface during the post-annealing process.

The organic ammonium cations can be divided into alkylammonium and arylammonium categories. The alkylammonium cations have a long chain shape, whereas the arylammonium cations feature a bulky hexagonal structure. The arylammonium cations impart aroma to the organic precursors, also called aromatic moieties. The alkylammonium cations also have a different name, called the aliphatic moieties. The hydrocarbon substituent on the ammonium cation can be tailored to achieve the required optoelectronic and hydrophobic effects. In this review, we collect different long or bulky organic ammonium cations for possible use as perovskite precursors to form a 2D perovskite layer on a 3D perovskite thin film. The following discussion will be divided into three sections to present a clear vision of the effects of hydrocarbon substituents. The first section will focus on linear or branched alkylammonium cations, the second section will focus on aromaticaryl ammonium cations with benzene rings, and the third section will focus on organic cations with oxygen (O), nitrogen (N), and sulfur (S) as substitutes for carbon (C) in pentagonal or hexagonal C-rings. Table 1, Table 2 and Table 3 summarize different PSCs passivated with different organic salts to form 3D/2D heterojunctions.

## 3. Alkylammonium Precursors

Alkylammonium is a family of organic salts containing aliphatic moieties. Table 1 lists the key photovoltaic parameters of 3D/2D bilayered PSCs with 2D capping layers based on the alkylammonium. This organic alkylammonium precursor has long hydrocarbon chains and loosely bound halogens. After the deposition of the aliphatic perovskite precursor solutions, the long cations remain on the surface and passivate the defects. In addition to effective surface passivation, the long organic spacer acts as an insulator between the 3D perovskite and the HTL. The 2D capping layer is made too thin so that the holes easily transfer from the 3D perovskite to the HTL. The 2D capping layer also acts as a humidity barrier, which effectively suppresses water molecule diffusion and increases stability against ambient moisture.

The ability to form a 2D capping layer on top of the 3D perovskite depends on the halide components (Cl, Br, or I). The halide component of the alkylammonium is usually Cl, Br, or I. The halide component can diffuse into the 3D perovskite lattice of the perovskite thin film. Arafat et al. [58] found that the diffusion of halide ions into the bulk perovskite does not depend on the crystallization process of the 2D perovskite. The halides diffuse rapidly during the deposition of the 2D precursor solution. Among octylammonium chloride/bromide/iodide, they obtained champion performance for the chloride one in iodine-based PSCs [58]. While selecting the appropriate halide component for alkylammonium perovskite precursors, Liu et al. [37] also found that the chloride-containing one demonstrates superior crystallographic properties, improved carrier transport, and better extraction compared to the bromide and iodide analogs in iodine-based perovskite.

The role of the aliphatic cation is beneficial in assisting the passivation of surface defects and in controlling the morphology of the 3D perovskite thin film. Defects located within grain boundaries or on the thin film surface degrade the solar cell performance. The surface defects produce interfacial defects between the active perovskite layer and HTL. These defects originate from organic cation vacancies or halide vacancies. Alkylammonium halide is a promising candidate and has been frequently used to passivate these defects. Yanoing et al. [35] found that the hydrocarbon chain length of aliphatic ammonium iodide is a crucial factor in the defect passivation effect. The hexylammonium iodide is more efficient in decreasing interfacial defects compared to butylammonium iodide. The hexylammonium iodide remarkably enhanced the photoluminescence lifetime and suppressed the interfacial charge recombination process. Due to the long hydrocarbon chain, strong polarity occurs in the organic molecule. Fu et al. [59] found that di-isopropylammonium chloride exhibits a large spontaneous polarization and possesses ferroelectric properties. Alkylammonium halide is also useful to regulate the morphology of the 3D perovskite thin film. Xin et al. [27] used diethylammonium bromide in planar PSCs to post-treat the MAPbI_3_ thin film. They observed the secondary growth of small crystals into larger ones, which led to large-grain formation in the 3D perovskite film. At the same time, there was a 2D capping layer on the MAPbI_3_ thin film surface. The 2D capping layer also enhanced the stability against moisture and light of the 3D perovskite thin film.

Aliphatic ammonium halides can also bear multiple functionalities, such as ethanol and methoxy groups. These additional functionalities can enhance the performance of PSCs by providing more interaction with the perovskite surface through hydrogen-to-halogen bonding, resulting in reduced charge recombination at the corresponding interface due to improved contact.

A cyclic aliphatic ammonium contains at least one all-carbon ring, which may be either saturated or unsaturated, but does not have an aromatic character. Jeong et al. [40] employed cyclohexylammonium and cyclohexylmethylammonium between perovskite and HTL in a 3D/2D configuration. They obtained PCEs of over 23% for cyclohexylammonium and over 24% for cyclohexylmethylammonium. The stability also improved by 4% for cyclohexylmethylammonium compared to cyclohexylammonium-based 3D/2D PSCs.

## 4. Arylammonium Precursors

Arylammonium is another family of organic cations that contain aromatic moieties. Table 2 lists the PSCs with their key photovoltaic parameters, which utilize different bulky arylammonium cations as a 2D capping layer. Among different arylammonium precursors, the phenylammonium, benzylammonium, and phenethylammonium precursor series are novel organic perovskite precursors for developing a 2D perovskite capping layer. The aromatic substituents are generally bulky *π*-conjugated hexagonal molecules with band gaps typically larger than 3.0 eV. Therefore, arylammnium is suitable as a spacer cation between 3D perovskite and HTL. The spacer effect is similar to that of aliphatic ammonium halide passivators.

The chemical and physical properties of the arylammonium precursor series vary depending on the hydrocarbon chain length attached to the ammonium group. Notably, the hydrocarbon chain between the aromatic ring and the ammonium halide is essential due to the *π*-electron delocalization effect of the ammonium halide. The lowest hydrocarbon chain length belongs to phenylammonium, and the highest belongs to phenethylammonium. The ability to form a 2D layer on the 3D perovskite layer also changes with the hydrocarbon chain length. For example, Li et al. [41] deposited phenylammonium iodide, phenylethylamine (benzylammonium) iodide, and phenethylammonium iodide between the perovskite and HTL and found phenylammonium iodide to be the best choice.

**Figure 3:**
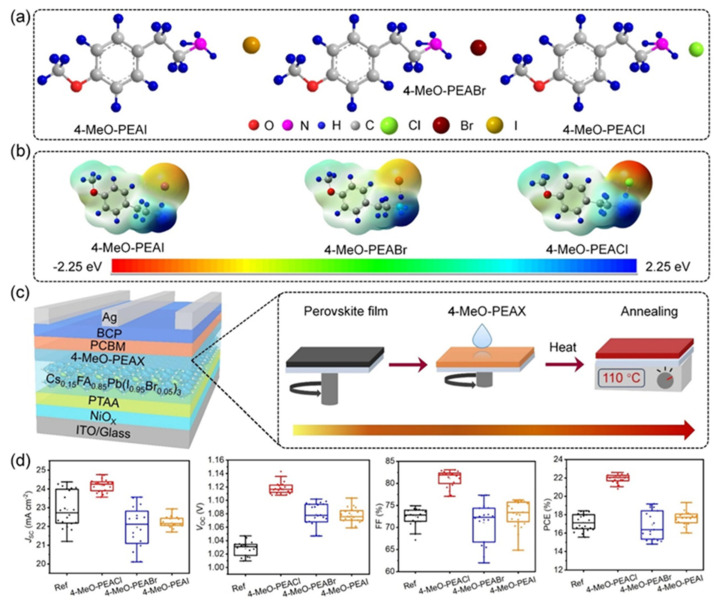
(**a**) The molecular structures of 4-methoxyphenethylammonium iodide/bromide/chloride. (**b**) Electrostatic maps of the above precursor salts. (**c**) Schematic diagram of the PSC architecture along with the preparation process. (**d**) Performance statistics of the key photovoltaic parameters for the fabricated PSCs with the above passivators. Reprinted with permission from Ref. [52].

The chemical and physical properties of the arylammonium precursors can also vary with the presence of different halides or groups at position four of the hexagonal benzene ring. When halides are present at position four, both the halides, including that at position four of the benzene ring, diffuse into the bulk of the 3D perovskite because halides are loosely bound to the bulky cation. This bulk modification is fully compatible with the bulk modification of the perovskite active layer by ionic liquids [60,61,62,63]. Howlader et al. [44] optimized the mixed 4-chloro-benzylammonium chloride and 4-chloro-benzylammonium bromide to passivate the bulk defects of the 3D perovskite film. Like alkylammonium, the PCE of PSCs also depends on the halide component of the arylammonium. Yang et al. [52] passivated the 3D perovskite thin film surface with 4-methoxyphenethylammonium iodide/bromide/chloride and obtained the best PCE with chloride. Figure 3a shows the ball-and-stick models of the three different passivators. Figure 3b shows the electrostatic maps of those precursors. We see that the dipole effect is most potent in 4-methoxyphenethylammonium chloride. The schematic diagram of the solar cell’s structure and its fabrication process are shown in Figure 3c. The data variation in the open-circuit voltage, short-circuit current density, fill factor, and PCE of the corresponding cells is presented in Figure 3d. We see that 4-methoxyphenethylammonium chloride passivated the PSC scores most in all four key parameters.

Besides halides, methyl, hydroxyl, methoxy, and ammonium are mentioned among various groups. The presence of these groups in position four of the arylammonium spacer cations alters the polar character of the bulky cation, as the electron-donating property of these groups increases the electron density of the ammonium. Thus, a strong molecular dipole is created between the bulky cation and halide, which improves the formation of a 2D perovskite capping layer. This leads to better 2D film formation with full coverage and larger grains. The methyl, hydroxyl, methoxy, and ammonium groups also have a role in enhancing the ability of the organic precursor solution to achieve a more homogeneous spatial distribution on the surface of the 3D perovskite thin film. Consequently, the 2D perovskite film has a more homogeneous energy landscape and significantly improves the transport of carrier counts between the 3D perovskite and HTL [50].

The ammonium, methyl, hydroxyl, and methoxy groups in position four of the bulky organic cation also enhance the lipophilic character, thereby improving the hydrophobic nature of the 2D perovskite film. At the same time, the ultrathin hydrophobic barrier layer formed after passivation also shields the 3D perovskite film surface from moisture ingress and environmental degradation, leading to the improved stability of the PSCs [64]. For example, 1,4-benzene diammonium iodide containing 2D perovskite-based PSCs showed remarkable stability under continuous illumination (could retain 95% PCE after 7000 h) and heating (could retain 95% PCE after 100 h at 80 °C) [65]. These groups also make the 2D perovskite hydrophobic. Nega et al. [66] added a trace amount of water that promoted the crystal formation of 4-methoxyphenylammonium lead iodide.

**Figure 4:**
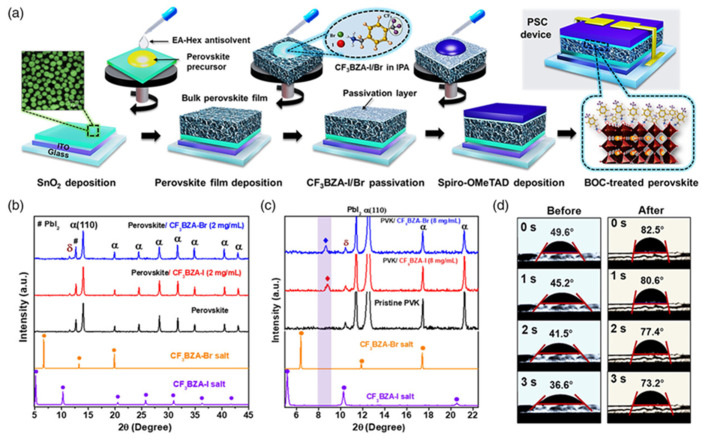
(**a**) Schematic illustration of the fabrication process of 3D/2D bilayered PSCs, where 4-trifluoromethyl benzylammonium iodide/bromide is used as a surface passivator. (**b**,**c**) XRD patterns of passivated perovskite films showing intense 2D perovskite peaks. (**d**) Water contact angle portfolio of perovskite film before and after passivation treatment with 4-trifluoromethyl benzylammonium bromide. Reprinted with permission from Ref. [45].

Again, fluorinated, bulky organic molecules show a higher affinity for acceptor-type defects on perovskite grain surfaces due to the highly electronegative nature of fluorine. In a study, Lin et al. [67] found that 4-trifluoromethyl-phenylammonium displayed an effective perovskite surface–passivator interaction compared to phenethylammonium. Zhu et al. [46] also observed that fluoromethylated modifier (4-trifluoromethyl benzylammonium iodide) has amphiphilic properties and mitigates the interfacial defects towards a stable PSC with a PCE near 24%. Tin (Sn)-based perovskites are prone to instability in ambient conditions due to oxidation, which creates Sn vacancies within the Sn-based perovskite. The fluoromethylated modifier can boost the PCE of Sn-based PSCs by passivating the defects in both the bulk and surface, soothing the oxidation of Sn and enhancing stability [68]. Figure 4a shows the 2D perovskite capping layer formation after the deposition of 4-trifluoromethyl benzylammonium iodide/bromide perovskite precursor on top of the 3D perovskite thin film. Figure 4b shows the X-ray diffraction of a 2D perovskite thin layer. The 4-trifluoromethyl benzylammonium iodide/bromide-treated 3D perovskite thin films offer firm peaks related to 2D perovskite. Figure 4c shows the water contact angle with the perovskite thin film before and after passivation. It is found that the water contact angle increases after 4-trifluromethyl benzylammonium bromide treatment, which means that the perovskite thin film becomes more hydrophobic [45].

## 5. Other Organic Precursors

Some organic precursors are very similar to arylammonium precursors. The arylammonium cation contains a benzene ring, while these organic cations contain N, O, or S in their hexagonal or pentagonal C-ring. In Table 3, we list the PSCs that used as much of a variety of organic cations as a 2D capping layer with their key photovoltaic parameters.

**Figure 5:**
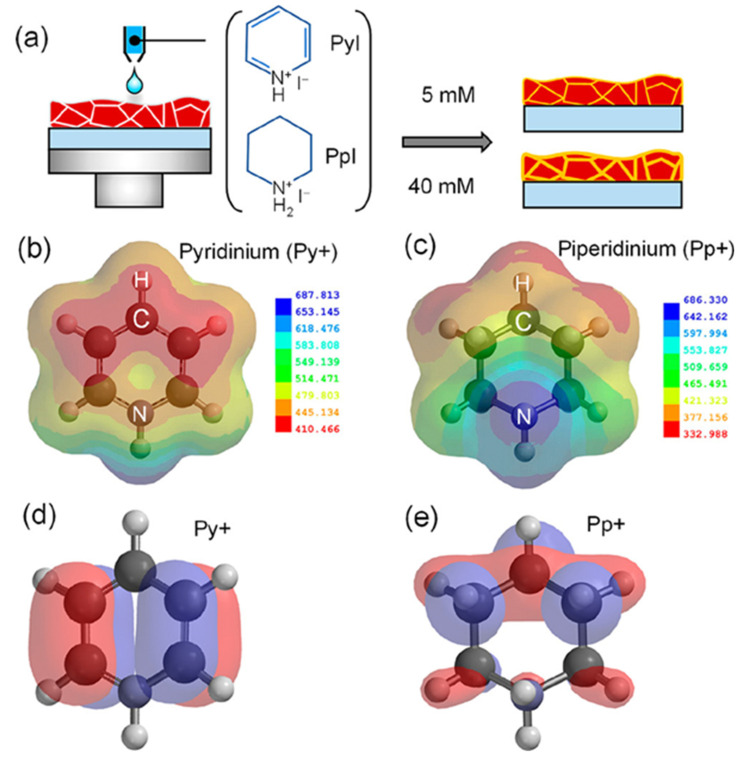
(**a**) The cartoon visualization of the procedure of 2D capping layer formation during the annealing of the perovskite thin film with pyridinium and piperidinium. ESP map of (**b**) pyridinium and (**c**) piperidinium. The ESP map shows electron-rich and electron-poor regions represented in red and blue colors, respectively. The space-filling model for the HOMO of (**d**) pyridinium and (**e**) piperidinium. Reprinted with permission from Ref. [53].

Yitian et al. [53] used pyridinium iodide for the surface defect passivation of the 3D perovskite thin film. They observed that the passivation mechanism differs from treatment with Lewis base pyridine, which can only bind to undercoordinated Pb ions. Here, the zwitterion molecule of pyridinium iodide can not only fill negatively charged iodine vacancies, but also interact with positively charged defects. They also observed that pyridinium passivation resulted in a smoother surface, lower defect densities, and non-radiative recombination in perovskite compared with the pyridine treatment. Kim et al. [54] compare and examine the effect of the chemical bonding nature of the organic precursors on the passivation. They took pyridinium iodide and piperidinium iodide as the passivators for the post-treatment of the 3D perovskite thin film. Pyridinium iodide has delocalized π electrons, and piperidinium iodide has no π bond and delocalized π electrons. They observed that the formation of a passivation layer depends on the concentration of the passivators. They also observed that different thicknesses for each organic perovskite precursor are required to achieve a decent PCE. Pyridinium required less thickness than piperidinium. Figure 5a shows a cartoon illustration of the post-treatment procedure of the perovskite thin film. The figure also shows the chemical structures of pyridinium iodide and piperidinium iodide. The figure shows a passivation layer formation within the grain boundaries and on the surface as a result of the post-treatment. The passivation layer thickness is also different. Figure 5b,c illustrate the electrostatic potential (ESP) maps of pyridinium and piperidinium cations. The colored illustration clearly shows that the electron density in pyridinium is relatively evenly distributed due to delocalized π electrons. On the other hand, due to the sp^3^ character of the bonding between C and N, there are no delocalized π electrons on the N atom. Figure 5d,e exhibit the calculated space filling model for the highest occupied molecular orbital (HOMO) of each cation. The HOMO of pyridinium is symmetric on both sides of its backbone, while the HOMO of piperidinium is non-symmetric. Hence, the piperidinium ion confirms a more decisive dipole moment of 4.14 D than that of the pyridinium ion (1.88 D).

**Figure 6:**
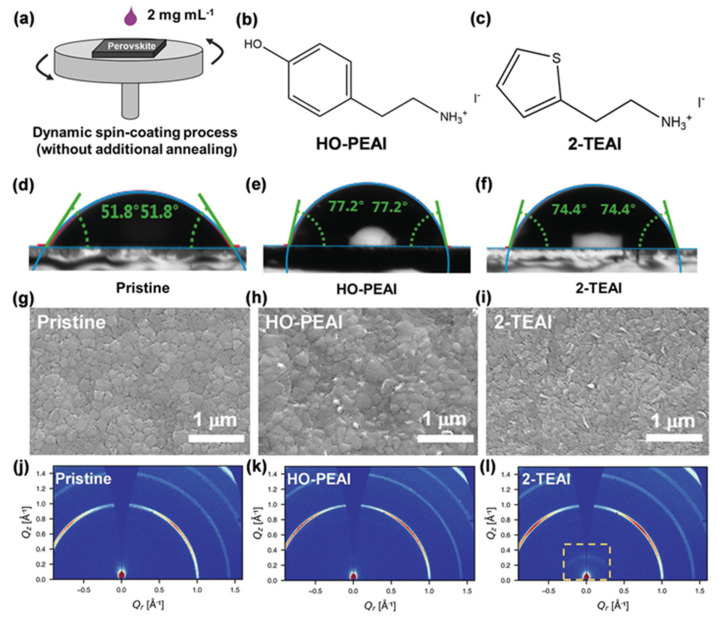
(**a**) Deposition process of 2D perovskite (HO-PEAI, 2-TEAI) on top of the 3D perovskite through a dynamic spin coating process without additional annealing. (**b**,**c**) Chemical structure of HO-PEAI and 2-TEAI. (**d**–**f**) Measurements of water contact angles after dropping water on the perovskite thin film surfaces. (**g**–**i**) Top-view SEM images on the same perovskite thin film surfaces. (**j**–**l**) Two-dimensional GIWAXS patterns from different samples. They were collected with an incidence angle of 0.3°. Reprinted with permission from Ref. [69].

Li et al. [69] found that thiopheneethylammonium facilitates the formation of a 2D structure on the perovskite thin film, compared to the hydroxyl-functionalized phenethylammonium (HO-PEA) moiety. Quasi-Fermi level splitting calculations and density functional theory disclosed that 2-thiopheneethylammonium iodide (2-TEAI) interacts more strongly with the perovskite surface. Thus, it obtained reduced non-radiative recombination at the perovskite/ETL interface and an improved V_oc_. Figure 6a shows the HO-PEAI/2-TEAI solution dipping and spinning procedure on top of the 3D perovskite. Figure 6b,c show the molecular structures of HO-PEAI and 2-TEAI. Figure 6d–f show the water contact angles on different perovskite surfaces. The angle is lower for 2-TEAI, which means it is more hydrophobic compared to HO-PEAI. Figure 6j–l shows the SEM images from the top surfaces of the pristine, HO-PEAI-treated, and 2-TEAI-treated perovskite thin films. We see clear grain boundaries from the top surface of the pristine perovskite thin film. After HO-PEAI treatment, the number of grains decreases. After 2-TEAI treatment, the grain boundaries almost vanished. Figure 6j–l show the 2D Grazing Incidence Wide-Angle X-ray Scattering (GIWAXS) patterns of different samples. The yellow square in Figure 6l indicates the weak scattering ring produced by the 2D structure and the prominent scattering in the 2-TEAI-treated perovskite thin film.

**Figure 7:**
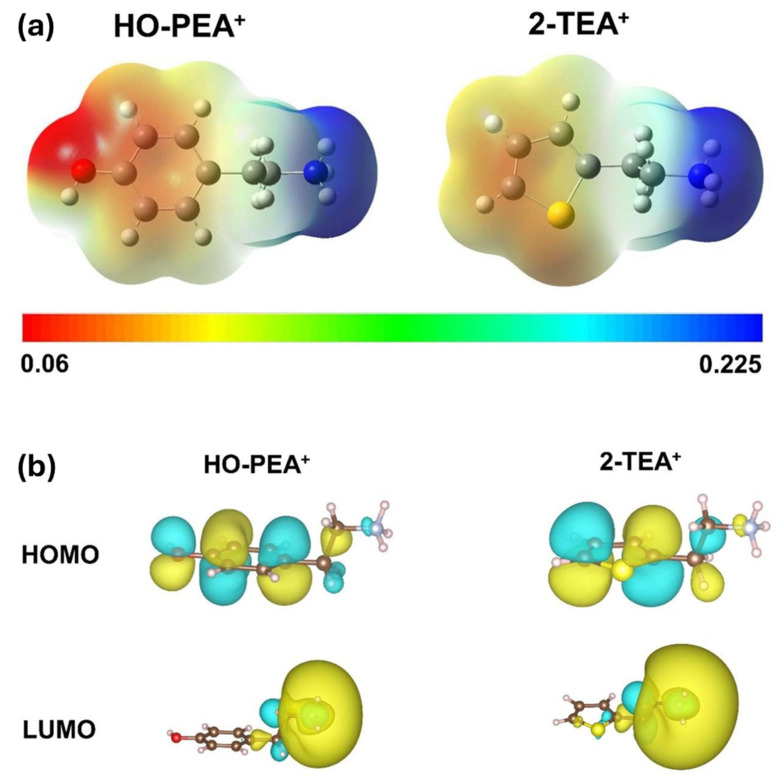
(**a**) The ESP maps of HO-PEA^+^ and 2-TEA^+^. (**b**) HOMO and LUMO of HO-PEA^+^ and 2-TEA^+^. Reprinted with permission from Ref. [69].

More details of the theoretical simulation from the above work by Li et al. [69] revealed that hydroxyl-functionalized phenethylammonium has a more even distribution of electrons. In contrast, the electron distribution is more uneven in thiopheneethylammonium. Therefore, the thiopheneethylammonium ion shows a more decisive dipole moment than the hydroxy-phenethylammonium ion. Figure 7a shows the ESP maps of hydroxyl-functionalized phenethylammonium and thiopheneethylammonium cations. The electron density in hydroxyl-functionalized phenethylammonium is relatively delocalized, while the electron localization is exhibited on the sulfur atom in thiopheneethylammonium. The space filling model for the HOMO and lowest occupied molecular orbital (LUMO) are presented in Figure 7b. The HOMO and LUMO of thiopheneethylammoniumis are more localized on sulfur atoms. Therefore, thiopheneethylammonium offers an intense dipole moment.

**Figure 8:**
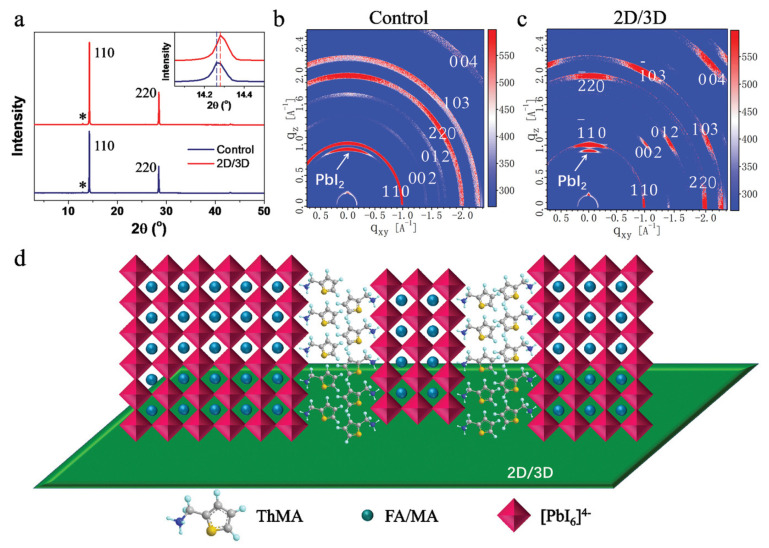
(**a**) XRD patterns of 3D and 3D/2D perovskite films. Asterisks symbolize the key reflections from PbI_2_. (**b**,**c**) GIWAXS of 3D and 3D/2D perovskite film. (**d**) Cartoon representation of the 3D/2D perovskite structure, where the 2D layer randomly grows on the 3D perovskite. Reprinted with permission from Ref. [55].

The 2D perovskite formation on top of the 3D perovskite thin film upon deposition of the organic perovskite precursor is a self-crystallization process. Therefore, the 2D perovskite film has a random crystalline orientation on top of the 3D perovskite film. Figure 8a shows the XRD peak related to 3D/2D perovskite. The evolution of 2D peaks in GIWAXS is demonstrated in Figure 8b,c. The crystallization process of the 2D perovskite on top of the 3D perovskite is random, and it can occur either vertically or horizontally to the substrate (see Figure 8d). That is why the 2D perovskite film can perform a multifunctional role, such as passivating the traps between the 3D perovskite layer and HTL, as well as facilitating the charge carrier transfer [42].

## 6. Effects on Efficiency

### 6.1. Reduced Charge Recombination

**Figure 9:**
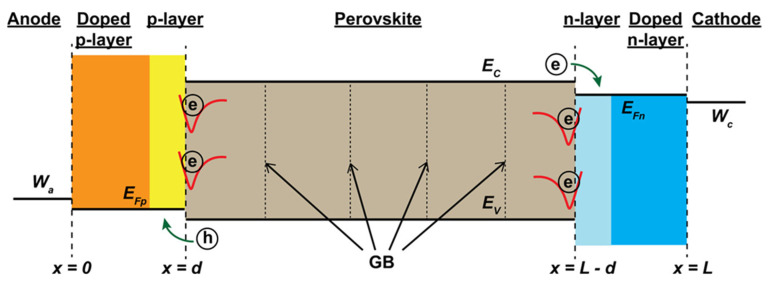
Schematic diagram of PSC illustrating the energy levels, interfacial trap states (red). Perovskite is an ambipolar semiconductor; therefore, the 2D perovskite can be considered an interfacial passivator (positive/negative type). Reprinted with permission from Ref. [72].

The defects on the surface generate shallow trap states, while the defects in the bulk generate deep trap states in the electronic band structure of perovskite. Both types of trap states enhance non-radiative photo-generated charge recombination. Non-radiative recombination significantly reduces electron or hole collection. Thus, the PCE of the PSC falls substantially. In Figure 9, photogenerated electrons are trapped at the interface due to trap states. In 3D/2D perovskite, the organic ammonium forms a 2D perovskite layer, mitigating the defects from the surface and bulk of the 3D perovskite, which helps reduce charge recombination [70]. At the same time, it works as an ion hunter during the formation of a 2D perovskite layer, thus inhibiting ions from diffusing to the adjacent layers. Different phenomena occur after the deposition of the organic ammonium, leading to the formation of a 2D capping film. The large organic cations remain on the surface of the 3D perovskite film as they cannot diffuse into the bulk [71]. The loosely bound halide ions of the organic perovskite salts diffuse into the bulk due to their small size. Suppose non-reacted metal halides exist in the 3D perovskite. In that case, there is a possibility of them interacting with the organic perovskite salt at the surface, forming a 2D perovskite capping layer.

### 6.2. Energy Level Alignment

The 2D capping layer on the 3D perovskite also works as an interface layer between the perovskite layer and the HTL. Two-dimensional perovskite has a higher band gap than 3D perovskite. Two-dimensional perovskite also has a deep Fermi level compared to 3D perovskite, near the valence band. The valence band maximum (VBM) does not change much in the 2D perovskite compared to in the 3D perovskite, while a significant difference occurs in the conduction band minimum (CBM). The CBM is less deep in the 2D perovskite than in the 3D one. After the 2D capping layer formation on the 3D perovskite, the Fermi level shifts towards the VBM of the overall 3D/2D perovskite. This is favorable for hole transport, so 2D capping layer formation is suitable at the interface of perovskite and HTL. It also blocks the electron transport from perovskite to HTL due to their high CBM difference and reduces the back recombination.

### 6.3. Reduced Ion Movement

The halide ions, originating from a deformed crystalline structure, could move around in the 3D cage. The 2D layer physically blocks the ion movement at the interface of the perovskite and HTL. Otherwise, the 2D layer embeds them and dynamically self-transforms into a 2D perovskite. The judicious choice of the 2D constituent is a prerequisite that opens up a new path for perovskite interface passivation. Sutanto et al. [57] examined 2-thiophenemethylammonium iodide (2-TMAI), 3-thiophenemethylammonium iodide (3-TMAI), and 2-thiopheneethylammonium iodide (2-TEAI) while forming 2D perovskite on 3D perovskite thin film. They observed that 2-TMAI and 3-TMAI can embed small ions migrating from the bulk and immobilize them. This did not happen for the 2-TEAI, which only physically blocked the ion at the interface. Interestingly, 2-TMAI and 3-TMAI passivated the interface and did not transform into 2D perovskite irrespective of the position of the phenemethylammonium. In the case of 2-TEAI, due to having a long pheneethylammonium hydrocarbon, it transformed into the 2D phase. This phenomenon implied that long hydrocarbons, such as those found in 2-TEAI, are a prerequisite for forming a structurally robust 2D layer, paramount to controlling device stability.

## 7. Effect on Stability

### 7.1. Humidity Stability

**Figure 10:**
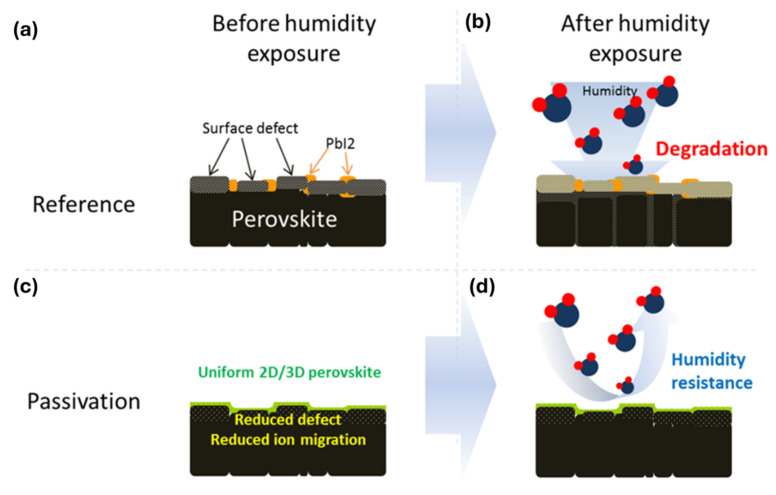
Schematic showing how the organic perovskite precursor passivation provides surface passivation and improves humidity stability. Reprinted with permission from Ref. [36].

Indeed, PSCs are still facing long-term stability concerns, which are barriers to commercialization. PSC stability depends on two significant perovskite issues, chemical decomposition and phase change, which are caused by interactions with environmental elements like light, heat, and moisture. The phase instability issue is inherent in perovskite material and is intimately related to the low activation energy of ion diffusion [73,74]. The low activation energy of vacancies encourages ion migration, for which the perovskite’s crystal lattice collapses, leading to the formation of an energetically stable non-perovskite phase. Different external stresses like light, moisture, heat, voltage, etc., trigger the transformation. Although 2D perovskite is relatively stable against water content in the air (humidity) due to its hydrophobic character of long or bulky organic cations, 3D perovskite is extremely sensitive to moisture. The black film gradually turns yellow when the 3D perovskite comes into contact with moisture. The water molecule slowly diffuses through the defects, like atomic vacancies or grain boundaries, and turns the perovskite into a hydrate form. Figure 10a shows the little presence of PbI_2_ within the surface defects. The surface defects and grain boundaries become yellowish after contact with humidity (see Figure 10b). The color of PbI_2_ is yellow, indicating the perovskite’s decomposition. Figure 10c,d show that the 3D/2D perovskite thin film remains black even after contact with humidity.

### 7.2. Thermal Stability

**Figure 11:**
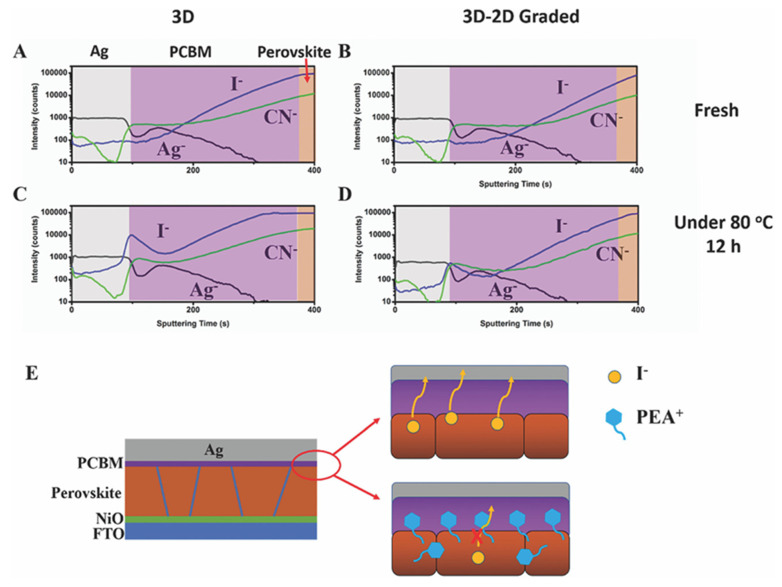
I^−^ ion migration phenomenon in the perovskite film is due to thermal stress. (**A**–**D**) TOF-SIMS depth profile of I^−^ and Ag^+^ ions of fresh and aged PSCs with 3D (**left**) and 3D/2D (**right**) under 80 °C for 12 h. (**E**) Schematic illustration of general ion diffusion in a 3D perovskite-based PSC (**top**) and suppressed ion diffusion in a 3D/2D-based PSC (**bottom**). Reprinted with permission from Ref. [49].

Long-term stability against thermal stress is another requirement for PSC commercialization. When the solar module is installed outside, the temperature increases by 25–30 °C compared to the surrounding environment. Due to the increase in module temperature, the solar cell temperature also increases, which is referred to as thermal stress. Long-term thermal stress is found to vaporize the MA^+^ cation in the organic–inorganic hybrid perovskite because the MA^+^ cation is volatile. Under continuous thermal stress, MA^+^ vaporizes and leaves vacancies in the perovskite matrix. In the perovskite matrix, unbounded halogen ions are prone to move freely. These free-moving halogen ions move toward the surface through these vacancies. The free halogen ions could diffuse through the HTL towards the back electrode. Sometimes, these halogen ions chemically react with the metal ions of the back electrode and produce metal halides. Metal halide formation harms the back electrode as it enhances its corrosion process. In a work by Bai et al. [49], they carried out depth-dependent time-of-flight secondary ion mass spectroscopy (TOF-SIMS) from both 3D and 3D/2D samples. TOF-SIMS was taken from both samples before and after heat treatment (see Figure 11). The I^−^ ion diffusion pattern through PCBM towards the Ag electrode shows similar characteristics in 3D and 3D/2D samples. However, they had higher I^−^ ion diffusion near the interface of PCBM and Ag in the 3D sample than in the 3D/2D sample after 12 h of continuous heating at 80 °C. This indicates the lower amount of I^−^ ion diffusion through PCBM towards the Ag electrode, as a large organic cation from 2D perovskite is a barrier to prevent it from diffusing.

## 8. Challenges

However, challenges remain in making a 3D/2D heterostructure. Here, we discuss some such challenges.

**Figure 12:**
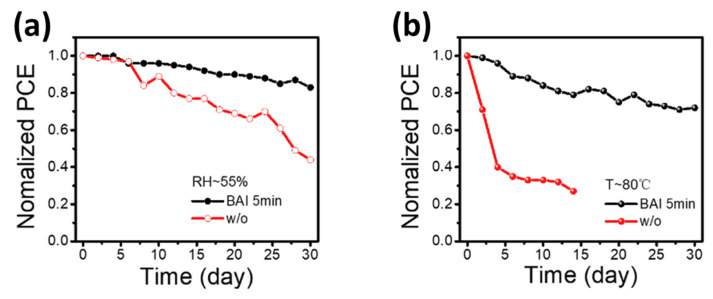
Vapor-processed 3D/2D perovskites. (**a**) Humidity stability, (**b**) thermal stability. Reprinted with permission from Ref. [31].

1. In most studies, isopropyl alcohol (IPA) is used as a solvent to make an organic ammonium halide solution, although it is not ideal for the 3D perovskite film. Instead of using IPA, the solvent-free method is developed, where the organic salts are deposited in the vapor phase to form a thin 2D capping layer on the surface of the 3D perovskite film. Through this process, Lin et al. [31] found better PSC stability against a relative humidity of 55% (see Figure 12a) and temperature of 80 °C (see Figure 12b).

**Figure 13:**
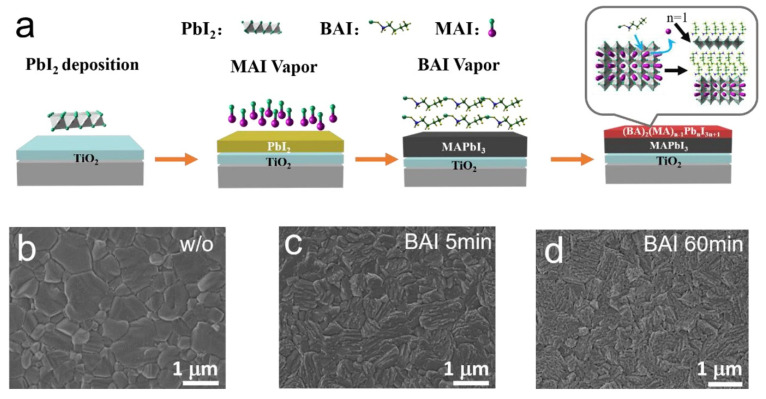
(**a**) Schematic diagram of the four-step growth mechanism of the 3D/2D perovskite structure through the vapor deposition process. SEM images from the top surfaces of the perovskite thin films (**b**) without, (**c**) with 5 min, and (**d**) 60 min BAI vapor treatment. Reprinted with permission from Ref. [31].

Figure 13a illustrates the vapor deposition process used to develop the extremely thin 2D perovskite film on top of the 3D perovskite film. First, PbI_2_ is deposited, followed by the methylammonium iodide (MAI) vapor that flows on top of the PbI_2_ thin film to prepare the MAPbI_3_ thin film. Then, butylammonium iodide (BAI) vapor is applied to the MAPbI_3_ thin film. Figure 13b–d show SEM images of the bare 3D perovskite thin film and the 3D/2D bilayered perovskite film after 5 and 60 min of BAI vapor deposition. It is noted that after 5 min, an extremely thin 2D film can be developed. If the vapor deposition lasts longer, such as 60 min, the 2D film converts into a 3D film.

2. Most studies find that halide ions diffuse immediately into the bulk because they are smaller and loosely bonded to the alkyl/arylammonium. Long or bulky organic cations also diffuse into the bulk under long-term thermal stress [75]. In long alkylammonium cations, the diffusion depends on the hydrocarbon chain length. Alan et al. [76] found that the 2D perovskite on top of the 3D perovskite thin film eventually transformed into a 3D perovskite under long-term thermal stress or illumination. They found that this transformation is slower for dodecylammonium compared to butylammonium. This indicates that the stability of the 3D/2D structure increases with the hydrocarbon chain length. Additionally, they found that the transformation process is even slower for 1,12-dodecanediammonium, suggesting that using diammonium alkaline ligands may be a possible pathway for forming a stable 3D/2D perovskite structure. In the case of arylammonium cations, it is challenging to prevent their diffusion into the bulk because they are smaller in size compared to alkylammonium cations. Park et al. [77] observed that fluorinated arylammonium offers better interfacial passivation and minimizes ligand intercalation reactivity with perovskites.

## 9. Conclusions

PSCs are showing promising performance as third-generation photovoltaics. Despite remarkable improvements in efficiency, crossing the stringent requirements concerning degradation remains a challenge. Therefore, significant endeavors are presently focused on tackling environmental degradation mechanisms. One way to address this limitation is by exploring interface engineering with organic perovskite materials, especially at the top interface near the back contact. Due to their low expense and adjustable electrochemical properties, passivation with organic ammonium halide precursors has emerged as a promising method for enhancing the stability of PSCs. This review sums up the latest research evolution in this area. It summarizes recent progress regarding organic ammonium halides and their critical functions in defect-healing procedures and upgrading the stability of PSCs. The organic ammonium halide passivators significantly improve efficiency and stability by reducing the recombination centers and contributing immunity against humidity resistance. Still, there is plenty of scope for additional investigations to understand the underlying mechanisms better and improve deposition methods to achieve the commercialization goals of PSC technology.

## Figures and Tables

**Table 1:** Key photovoltaic parameters of PSCs with 3D/2D perovskite heterojunctions. The 2D layers are based on alkylammonium cations with linear or branched chains.

Chemical Name (2D Layer)	Structural Formula (2D Layer)	Chemical Formula (3D Layer)	V_oc_ (V)	J_sc_ (mA/cm^2^)	FF (%)	PCE (%)	Stability (Hours)	Ref.
Ethylammonium	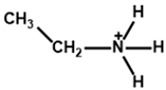	FA_0.9_Cs_0.07_MA_0.03_Pb(I_0.92_Br_0.08_)_3_	1.12	24.36	80.4	22.30	550 (90%)	[28]
		Cs_0.05_FA_0.80_MA_0.15_Pb(I_0.85_Br_0.15_)_3_	1.12	21.81	75	18.92	-	[29]
Propylammonium	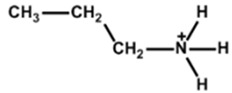	Cs_0.05_FA_0.80_MA_0.15_Pb(I_0.85_Br_0.15_)_3_	1.13	22.26	78	20.25	-	[29]
Butylammonium	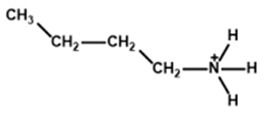	Cs_0.05_FA_0.80_MA_0.15_Pb(I_0.85_Br_0.15_)_3_	1.11	22.01	76	19.43	-	[29]
		(FAPbI_3_)_0.92_(MAPbBr_3_)_0.08_	1.14	24	79.90	22	2400 (90%)	[30]
		MAPbI_3_	1.02	23.37	68.70	16.50	7200 (74%)	[31]
		MAPbI_3_	1.16	20.89	80.40	19.48	600 (92%)	[32]
		Cs_0.07_Rb_0.03_FA_0.765_MA_0.135_PbI_2.55_Br_0.45_	1.20	23.94	79.31	22.77	20 (90%)	[33]
		Cs_0.05_(MA_0.17_FA_0.83_)Pb(I_0.83_Br_0.17_)_3_	1.06	19.40	76.69	15.74	100 (86%)	[34]
		Cs_0.07_FA_0.79_MA_0.14_Pb(I_0.88_Br_0.12_)_3_	1.12	22.95	75	19.43	1200 (99%)	[35]
Hexylammonium	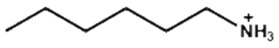	(FAPbI_3_)_0.92_(MAPbBr_3_)_0.08_	1.15	24.20	80.30	22.40	2400 (95%)	[30]
		Cs_0.07_FA_0.79_MA_0.14_Pb(I_0.88_Br_0.12_)_3_	1.14	23.76	76	20.62	1200 (99%)	[35]
Octylammonium	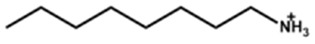	(FAPbI_3_)_0.92_(MAPbBr_3_)_0.08_	1.15	24.20	79.80	22.10	2400 (99%)	[30]
		Cs_0.05_(MA_0.17_FA_0.83_)Pb(I_0.83_Br_0.17_)_3_	1.02	19.37	76.70	15.19	-	[34]
Iso-Butlammonium	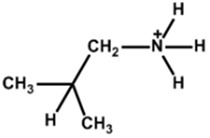	(FAPbI_3_)_0.85_(MAPbBr_3_)_0.15_	1	-	-	21.7	912 (87%)	[36]
Tert-Pentylammonium	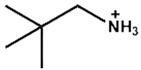	FA(MA)PbI_3_	1.16	24.71	81.62	23.35	1500 (95%)	[37]
Diethylammonium	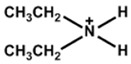	MAPbI_3_	1.06	21.93	78.70	18.30	60 (90%)	[27]
2-Methoxyethylammonium	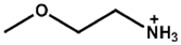	(FAPbI_3_)_1–x_(MAPbBr_3–y_Cl_y_)_x_	1.08	23.79	69.07	17.74	-	[38]
Guanidinium	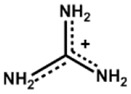	FA_0.9_Cs_0.07_MA_0.03_Pb(I_0.92_Br_0.08_)_3_	1.106	24.45	75.3	20.90	550 h (90%)	[28]
		Cs_0.07_MA_0.14_FA_0.79_Pb(I_0.83_Br_0.17_)_3_	1.2	22.8	75.1	18.2	-	[39]
Cyclohexylammonium	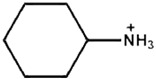	FAPbI_3_	1.13	24.72	83.06	23.10	200 s (93%)	[40]
Cyclohexyl-methylammonium	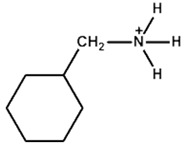	FAPbI_3_	1.14	24.83	84.25	23.91	200 s (97%)	[40]

All the structural formula images are taken from www.greatcellsolarmaterials.com (accessed on1 June 2025).

**Table 2:** Key photovoltaic parameters of PSCs with 3D/2D perovskite heterojunctions. The 2D layers are based on arylammonium cations with bulky structures.

Chemical Name (2D Layer)	Structural Formula (2D Layer)	Chemical Formula (3D Layer)	V_oc_ (V)	J_sc_ (mA/cm^2^)	FF (%)	PCE (%)	Stability (Hours)	Ref.
Phenylammonium	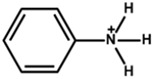	Cs_0.05_FA_0.79_MA_0.16_PbI_2.4_Br_0.6_	1.18	21.19	83	21.33	550 h (70%)	[41]
Benzylammonium	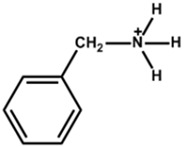	Cs_0.08_FA_0.77_MA_0.12_PbI_2.62_Br_0.35_	1.08	24.48	78.80	20.79	600 h (80%)	[42]
		FA_0.15_Cs_0.85_Pb(I_0.73_Br_0.27_)_3_	1.24	19.83	73.70	18.13	960 h (80%)	[43]
4-Chloro-Benzylammonium	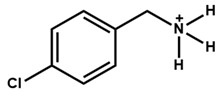	FA_0.6_MA_0.4_PbI_2.7_Cl_0.3_	1.12	25.69	72.78	21	672 h (88%)	[44]
4-Trifluoromethyl-Benzylammonium	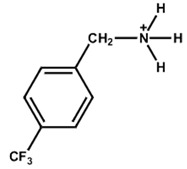	FA_0.85_MA_0.15_PbBr_0.45_I_2.55_	1.16	22.21	81.25	20.75	300 h (86%)	[45]
		Cs_0.05_FA_0.85_MA_0.10_Pb(I_0.97_Br_0.03_)_3_	1.16	24.98	82.40	23.94	500 h (96.5%)	[46]
Phenethylammonium	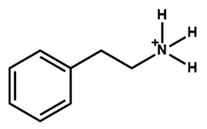	MAPbI_3_	1.06	21.80	76.60	17.70	-	[47]
		Cs_0.05_(FA_0.83_MA_0.17_)_0.95_Pb(I_0.83_Br_0.17_)_3_	1.11	73	22.89	18.91	1000 h (90%)	[26]
		Cs_0.1_FA_0.74_MA_0.13_PbI_2.48_Br_0.39_	1.14	24.20	76.60	21.15	1440 h (84%)	[48]
		MAPbI_3_	1.17	21.80	78	19.89	720 h (96%)	[49]
		Cs_0.1_FA_0.77_MA_0.13_PbI_2.59_Br_0.41_	1.14	23.43	77.4	20.62	-	
4-Chloro-Phenethylammonium	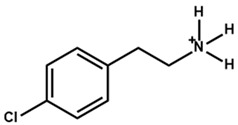	Cs_0.05_(FA_5/6_MA_1/6_)_0.95_Pb(I_0.85_Br_0.15_)_3_	1.15	23.68	85	23.07	33 h (90%)	[50]
4-Fluoro-Phenethylammonium	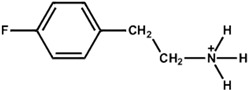	Cs_0.05_(FA_5/6_MA_1/6_)_0.95_Pb(I_0.85_Br_0.15_)_3_	1.16	24.13	84.60	23.72	33 h (90%)	[50]
		Cs_0.1_FA_0.77_MA_0.13_PbI_2.59_Br_0.41_	1.13	23.21	78.40	20.53	-	
4-Methyl-Phenethylammonium	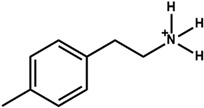	FAPbI_3_	1.10	24.76	78.6	21.4	-	[51]
		(FAPbI_3_)_1–x_(MAPbBr_3–y_Cl_y_)_x_	1.16	24.49	76.94	21.85	-	[38]
4-Methoxy-Phenethylammonium	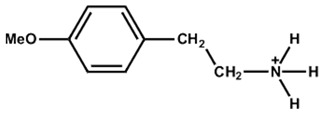	Cs_0.15_FA_0.85_Pb(I_0.95_Br_0.05_)_3_	1.13	24.33	82.31	22.63	1000 h (84%)	[52]
		(FAPbI_3_)_1–x_(MAPbBr_3–y_Cl_y_)_x_	1.18	25.04	77.79	22.98	1000 h (89%)	[38]

All the structural formula images are taken from www.greatcellsolarmaterials.com (accessed on 1 June 2025).

**Table 3:** Key photovoltaic parameters of PSCs with 3D/2D perovskite heterojunctions. The 2D layers are based on other organic cations with bulky structures with O, N, or S elements.

Chemical Name (2D Layer)	Structural Formula (2D Layer)	Chemical Formula (3D Layer)	V_oc_ (V)	J_sc_ (mA/cm^2^)	FF (%)	PCE (%)	Stability (Hours)	Ref.
Pyridinium	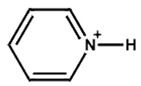	Cs_0.05_(MA_0.17_FA_0.83_)_0.95_Pb(I_0.83_Br_0.17_)_3_	1.19	23.12	78	21.42	720 h (81.40%)	[53]
		FA_0.9_Cs_0.1_PbI_2.8_Br_0.2_	1.15	23.70	81.20	22.26	0.5 h (115.01%)	[54]
Piperidinium	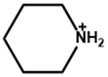	FA_0.9_Cs_0.1_PbI_2.8_Br_0.2_	1.15	23.58	80.49	21.77	0.5 h (99.40%)	[54]
Imidazolium	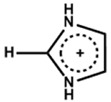	FA_0.9_Cs_0.07_MA_0.03_Pb(I_0.92_Br_0.08_)_3_	1.10	24.14	79.40	21.60	550 h (90%)	[28]
2-Thiophenemethylammonium	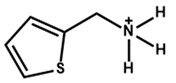	CH_3_NH_3_PbI_3_	1.16	22.36	81	21.49%	1680 h (99%)	[55]
		-	1.09	21.45	80.31	18.58	720 h (98)	[56]
		[(FAPbI_3_)_0.87_(MAPbBr_3_)_0.13_]_0.92_(CsPbI_3_)_0.08_	1.13	23.50	75.10	19.97	1000 h (70%)	[57]
2-Thiopheneethylammonium	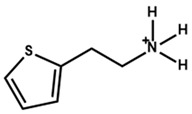	Cs_0.05_FA_0.79_MA_0.16_PbBr_0.6_I_2.4_	1.2	21.93	83	21.09	-	[55]
		[(FAPbI_3_)_0.87_(MAPbBr_3_)_0.13_]_0.92_(CsPbI_3_)_0.08_	1.12	23.60	76.50	19.42	1000 h (90%)	[57]

All the structural formula images are taken from www.greatcellsolarmaterials.com (accessed on 1 June 2025).
